# Profil épidemio-clinique et échographique des moles hydatiformes à Abidjan

**DOI:** 10.11604/pamj.2019.33.264.17400

**Published:** 2019-07-29

**Authors:** Kouamé N’goran, Kouadio Kouamé Eric, Doukouré Brahima, Sétchéou Alihonou, Konan Anhum Nicaise, Ettien Kouamé Jean-Jacques, N’Goan-Domoua Anne-Marie, Konan Alexis Victorien

**Affiliations:** 1 Service de Radiologie, CHU de Yopougon, Abidjan, Côte d’Ivoire; 2 Service de Radiologie CHU de Treichville, Abidjan, Côte d’Ivoire; 3 Service d’Anatomo-pathologie CHU de Cocody, Abidjan, Côte d’Ivoire; 4 Hôpital Mère-Enfant de Bingerville, Abidjan, Côte d’Ivoire

**Keywords:** Grossesse molaire, mole hydatiforme, métrorragie du 1er trimestre, Afrique, Molar pregnancy, hydatidiform mole, metrorrhagia of the 1st trimester, Africa

## Abstract

**Introduction:**

Décrire les aspects épidémio-cliniques et échographiques des môles hydatiformes (MH) à Abidjan.

**Méthodes:**

étude transversale de 6 ans (janvier 2011 à décembre 2016) réalisée au CHU de Yopougon au Service de Radiologie. Elle a consisté en la description du profil épidémio-clinique et échographique des patientes porteuses de MH. Les examens échographiques ont été réalisés par voie mixte (endovaginale et sus pubienne) en modes B et Doppler Couleur par des radiologues seniors. Une étude anatomo-pathologique du contenu utérin a été effectuée.

**Résultats:**

Vingt-cinq cas de MH ont été diagnostiquées sur 12,190 échographies obstétricales réalisées soit 0,2% d'incidence radiologique. L’âge moyen des patientes était de 33,4 ans avec des extrêmes de 22 et 50 ans. Il n'y avait pas de classe d'âge dominante. La notion de masse abdominale 36% et de métrorragies 28% étaient les signes cliniques prédominants qui accompagnaient l'aménorrhée (100%). Sur le plan échographique, l'utérus était hypertrophique dans 100% des cas, homogène dans 96% et myomateux dans 4% des cas. La MH avait une épaisseur moyenne de 42,7mm. Son aspect était décrit comme vésiculaire dans 68%, en nid d'abeille dans 16%, multikystique dans 12% et en tempête de neige dans 4%. Les MH étaient classées partielles dans 4% des cas, complètes dans 92% des cas et invasives dans 4% des cas. Les ovaires étaient hypertrophiques dans 44% des cas avec une notion de macrofollicules dans 32% et des kystes dans 8% des cas. Le diagnostic échographique de MH a été confirmé à l'anatomopathologie dans 100% des cas.

**Conclusion:**

Les MH demeurent rares à Abidjan et sont dominées par la forme complète. La notion de survenue à des âges extrêmes n'a pas été retrouvée.

## Abstract (en)

**Introduction:**

The purpose of our study was to describe the epidemioclinical and ultrasonographic features of hydatidiform moles (HM) in Abidjan.

**Methods:**

We conducted a cross-sectional study in the Department of Radiology, University Teaching Hospital of Yopougon over a 6-year period (January 2011-December 2016). In this study we describe the epidemioclinical and ultrasonographic profile of patients with HM. Ultrasonographic exams were performed using a mixed-methods approach (intravaginal and subpubic) based on B-mode and Color Doppler by senior radiologists. Anatomopathological examination of uterine content was performed.

**Results:**

Out of 12190 obstetric ultrasound performed, twenty-five cases of HM were diagnosed reflecting a radiological referral rate of 0.2%. The average age of patients was 33.4 years, ranging from 22 to 50 years. There was no dominant age class. The main clinical signs associated with amenorrhea (100%) included abdominal mass 36% and vaginal bleeding 28%. Ultrasound showed hypertrophic uterus in 100% of cases, homogeneous uterus in 96% of cases and myomatous uterus in 4% of cases. MH had an average thickness of 42.7 mm with vesicular appearance in 68% of cases, “honeycomb” appearance in 16%, multicystic appearance in 12% and snowstorm appearance in 4%. MHs were classified as partial in 4% of cases, complete in 92% of cases and invasive in 4% of cases. Hypertrophic ovaries were found in 44% of cases with macrofollicles in 32% of cases and cysts in 8% of cases. Ultrasonographic diagnosis of HM was confirmed by anatomopathological examination in 100% of cases.

**Conclusion:**

HMs are rare in Abidjan and are dominated by the complete hydatidiform mole. Its occurrence at the extreme ages wasn’t found.

## Introduction

Les môles hydatiformes (du grec môles: masse et hydatide: sac hydrique) appartiennent aux groupe des maladies trophoblastiques gestationnelles au cours desquelles le trophoblaste prolifère de manière anarchique et forme des vésicules hydropiques [1, 2]. Elles sont caractérisées par l'absence d'embryon et la dégénérescence villeuse totale du placenta, et résultent de la fécondation d'un ovule dont le pronucléus maternel est absent ou immature [2, 3]. Les môles hydatiformes (MH) représentent un réel problème de santé publique notamment dans les « pays du sud » et de l'Asie, de par leur incidence et leur risque d'évolution vers la môle invasive et le choriocarcinome [2]. Les modalités diagnostiques et de prise en charge étaient initialement basées sur l'histoire clinique et les données biologiques et histologiques. Actuellement, elles incluent en grande partie l'imagerie, notamment l'échographie et l'imagerie par résonnance magnétique [4]. À cause de sa survenue le plus souvent dans une population au niveau socio-économique bas [2], l'imagerie par résonnance magnétique ne peut pas être envisagée comme moyen diagnostique dans nos contrées. L'échographie s'avère être le seul moyen d'imagerie effectif dans sa prise en charge dans notre pratique quotidienne. C'est pourquoi, nous avons initié cette étude pour décrire les aspects échographiques rencontrées au cours des moles hydatiformes dans un pays d'Afrique subsaharienne ainsi que la population concernée.

## Méthodes

Nous avons réalisé une étude transversale de 6 ans (2011 à 2016) au Service de Radiologie du CHU de Yopougon. Elle a concerné toutes les échographies pelviennes ayant mis en évidence une MH dont certaines ont été confirmées par l'examen anatomopathologique du contenu utérin après aspiration. Les variables recherchées étaient l'âge, le motif de l'échographie, la taille de l'utérus, l'échostructure de l'utérus, l'aspect et le signal Doppler du contenu utérin ainsi que les ovaires. Ces variables étaient regroupées en paramètres épidémio-cliniques (fréquence, âge et indications) et échographiques (aspect de l'utérus et des annexes, le type de MH). Les échographies avaient été réalisées par voie mixte (endovaginale et sus pubienne) en modes B et Doppler Couleur. Les données ont été recueillies grâce aux comptes rendus d'échographie et au registre d'anatomopathologie.

## Résultats

**Figure 1:**
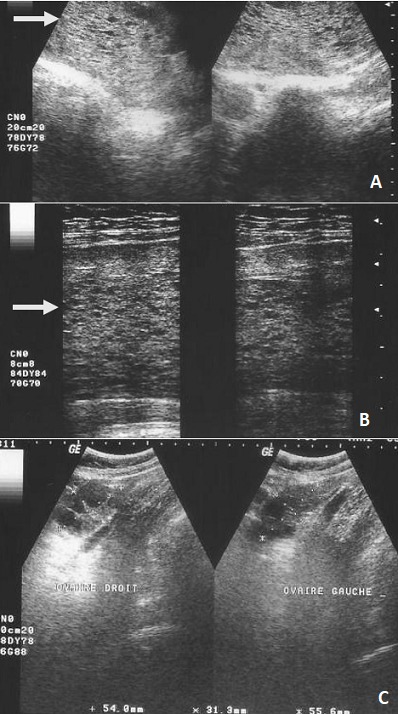
A) MH complète donnant un aspect multivésiculaire (flèche) vue avec une sonde convexe; B) linéaire; C) associée à de gros ovaires macrofolliculaires

**Figure 2:**
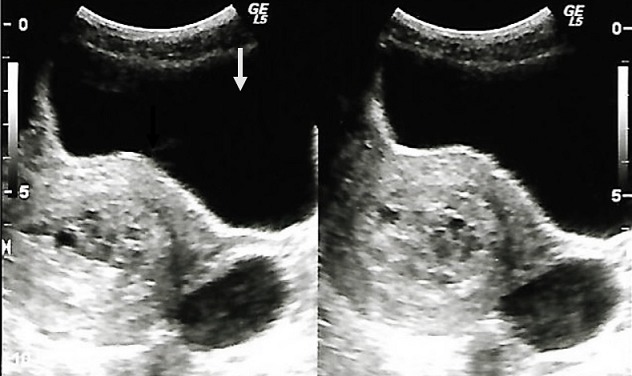
échographie pelvienne réalisée par voie sus pubienne chez une jeune dame de 28 ans présentant des métrorragies dans un contexte d’aménorrhée gravidique. Aspect en tempête de neige du contenu utérin (flèche blanche) avec un kyste bilatéral de l’ovaire (flèche noire)

**Figure 3:**
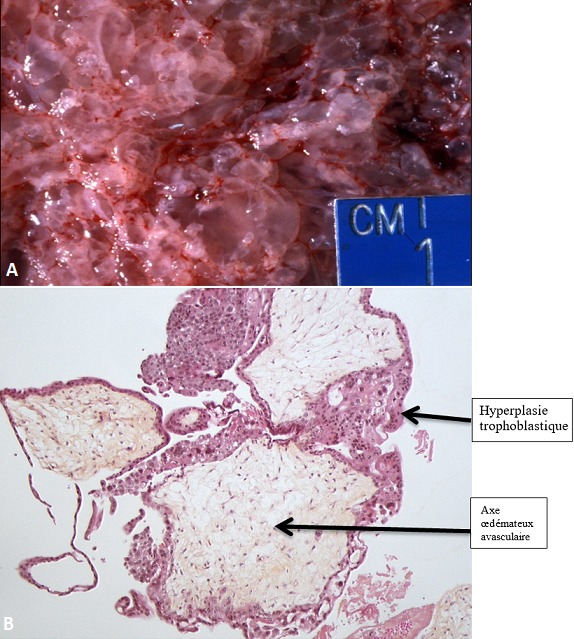
Aspect anatomopathologique du contenu utérin traduisant une môle hydatiforme; A) placenta avec de nombreuses vésicules de taille variable; B) hématoxyline-Eosine (HE) X100: villosités placentaires de taille variable avec un axe œdémateux avasculaire et une hyperplasie trophoblastique circonférentielle ou partielle

Nous avons objectivé 25 cas de MH sur 12,190 échographies obstétricales réalisées pendant la période d'étude (soit 0,2% d'incidence échographique). Les patientes étaient âgées de 22 à 50 ans avec une moyenne d'âge de 33,4 ans. Il n'y avait pas de classe d'âge prédominant (Tableau 1). Les indications des échographies étaient dominées par la masse abdominale (9 cas, 36%) suivies par les métrorragies (7 cas, 28%), les douleurs pelviennes (5 cas, 20%) et le diagnostic de grossesse (4 cas, 16%). La notion d'aménorrhée était retrouvée chez toutes les patientes (100%). Au plan échographique, l'utérus était hypertrophique avec les dimensions moyennes suivantes: hauteur = 138 mm (78 à 288 mm), largeur = 99 mm (51 à 126 mm) et épaisseur = 73 mm (42 à 96 mm). L'utérus était homogène dans 24 cas (96%) et myomateux dans 1 seul cas (4%). La MH avait une épaisseur moyenne de 42,7 mm (18 à 98 mm). Son aspect (Tableau 2) était décrit comme multivésiculaire (Figure 1) était prédominante (17 cas soit 68%), suivi de l'aspect en nid d'abeille dans 4 cas (16%). Les MH étaient classées partielles (Figure 2) dans 4% des cas, complètes (Figure 1) dans 92% des cas et invasives dans 4% des cas. Les ovaires étaient hypertrophiques dans 44% des cas avec une notion de macrofollicules dans 32% et des kystes dans 8% des cas. Dans 12 cas (48%), une étude anatomopathologique du contenu utérin a été effectuée (Figure 3). Elle a permis de confirmer la MH dans 100% des cas. Ce qui confère à l'échographie une valeur diagnostique positive de 100%.

**Tableau 1:** Répartition des patientes selon la classe d’âge

Classe d’âge	Effectif	Pourcentage (%)
20-24	5	20
25-29	5	20
30-34	5	20
35-39	5	20
> 40	5	20
**Total**	25	100

**Tableau 2:** Répartition des patientes selon l’aspect échographique de la MH

Aspect de la MH	Effectif	Pourcentage (%)
Multivésiculaire	17	68
Nid d’abeille	4	16
Multikystique	3	12
Tempête de neige	1	4
**TOTAL**	25	100

## Discussion

Sur un total de 12,190 échographies obstétricales réalisées pendant les 6 ans de notre étude, nous avons répertorié 25 cas de MH. Ce qui correspond à une incidence échographique de 0,2% de cas. Nos résultats étaient en concordance avec Tsuala au Cameroun [5] qui a mis en évidence 1 cas sur 556 grossesses en une année soit 0,18%. Mais ce taux est doublé en Afrique du nord où Boufettal au Maroc [2] a objectivé 0,4% de cas. Cette incidence est pratiquement insignifiante en Europe où Candelier [1] met en évidence moins de 1/1.000 grossesses. Cette différence de prévalence s'expliquerait par la survenue de cette affection en milieu socio-économique défavorisé. En effet selon Boufettal [2], les patientes concernées ont un niveau socio-économique bas, appartenant à des populations à risque soumises à des conditions climatiques drastiques et à un environnement agricole. Dans notre, la population concernée par les MH était relativement jeune et la notion de survenue aux âges extrêmes telles que décrites dans la littérature [2, 5] n'a pas été retrouvée. En effet, la moyenne d´âge des femmes était de 33, 4 ans avec des extrêmes de 22 et 50 ans. Les patientes étaient plus jeunes au Maroc avec une moyenne d'âge de 25 ans avec des extrêmes de 16 à 55 ans [2]. Il en était de même pour l'unique patiente de Tsuala au Cameroun [5] qui avait 23 ans. Dans notre étude il n'y avait pas de classe d'âge dominante contrairement à Boufetall chez qui les tranches d'âge de 20 à 29 ans (39,8 %) et 30 à 39 ans (29,5%) étaient prédominantes. Au plan clinique, la masse abdominale (36%) et les métrorragies (28%) étaient les signes d'appels prédominants qui accompagnaient la notion d'aménorrhée qui quant à elle était objectivée dans 100% des cas. Pour Tsuala [5], la métrorragie reste le signe majeur de la MH. Mais elle n'était présente que dans 93,7% des cas dans l'étude de Boufettal [2]. Selon la littérature [6], les signes cliniques pathognomoniques de la MH sont les métrorragies du 1^er^ trimestre associées à un utérus trop volumineux pour le terme. Quoi qu'il en soit, l'échographie constitue le moyen d'imagerie majeur pour rétablir le diagnostic [4, 7, 8]. L'aspect échographique caractéristique des MH [7] est celui d'une formation échogène et hétérogène, donnant un aspect en tempête de neige, en « nid d'abeille » ou multivésiculaire. L'association d'ovaires présentant plusieurs kystes lutéiniques est fréquente. L'aspect en « nid d'abeille » a été retrouvé dans 4 cas (16%) et en tempête de neige dans 4% des cas. L'aspect échographique le plus fréquemment décrit dans notre étude était l'aspect multivésiculaire (68%). Boufettal [2] a objectivé 87,5% d'image en « tempête de neige » et en « nid d'abeille ». Selon Chelli [4], l'aspect multivésiculaire était auparavant qualifié de « nid d'abeille » ou de « tempête de neige » sur les anciens appareils d'échographie qui manquaient de résolution spatiale suffisante pour distinguer les vésicules de petite taille. L'augmentation de la taille de l'utérus est un signe échographique important. Elle était retrouvée dans 100% des cas dans notre étude et dans 85% des cas dans celui de Boufettal [2]. Selon Lazrak [9], lorsque l'échographie montre des kystes dispersés dans le placenta et que le diamètre de l'utérus est augmenté, le diagnostic prédictif d´une môle partielle est estimé à 90% des cas. Dans notre étude, les dimensions de l'utérus pouvaient atteindre 28,8 cm de hauteur contre 22 cm dans celui de Tsuala [5]. Les moles hydatiformes sont habituellement classées en môle partielle et complète; les deux formes pouvant être invasive ou non invasive. Dans notre pratique nous les avons classés en MH partielles dans 4% des cas, complètes dans 92% des cas et invasives dans 4%. Les deux premières sont des formes bénignes de la maladie trophoblastique et la 3^ème^ forme peut être considérée comme potentiellement maligne. Lazrak [9] a décrit un cas de môle partielle invasive avec métastase pulmonaire au Maroc. Selon Delcominette en Suisse [10], la mole invasive a une évolution vers une néoplasie maligne qui s'observe dans 10 à 15% des cas des môles complètes et moins de 1% des môles partielles. En cas de doute, l'examen de l'environnement péri-utérin peut aider l'échographiste à asseoir le diagnostic. Les hypertrophies kystiques, le plus souvent bilatérales constituent un signe clé venant en appoint de l'augmentation de la taille de l'utérus et l'aspect multivésiculaire [7, 11-14]. Dans notre étude, les ovaires étaient hypertrophiques dans 44% des cas avec une notion de macrofollicules dans 32% et des kystes dans 8% des cas. Un accroissement du volume des ovaires a été objectivé dans 15 à 30% des cas au Cameroun [5] et dans 30,7% des cas, des kystes ont été retrouvé dans l'étude de Boufettal [2]. Les patientes ont bénéficié d'une aspiration du contenu utérin avec un examen anatomopathologique. Le diagnostic de MH a été confirmé dans 100% des cas y compris le cas de môle invasif. Cette valeur diagnostique positive de l'échographie était de 96% dans les môles complètes chez Chelli et 28% dans les cas de môles partielles [2]. Cette grande différence peut s'expliquer par les consultations tardives en Afrique subsaharienne rendant la tâche facile à l'échographiste qui rencontre le patient à une période où toute la sémiologie est présente et évidente contrairement aux diagnostics précoces effectués dans les pays développés [15].

## Conclusion

Les moles hydatiformes demeurent rares à Abidjan et sont dominées par la forme complète. Elles surviennent de façon équitable dans toutes les classes d'âge et sont révélées par une notion de masse pelvienne et de métrorragies dans un contexte d'aménorrhée. La forme complète est prédominante et l'aspect vésiculaire associé à une hypertrophie kystique des ovaires constituent les signes échographiques classiquement observés. L'imagerie médicale fait le diagnostic dans 100% des cas.

### État des connaissances actuelles sur le sujet

- La classification des môles hydatiformes;
- Les aspects en imagerie sont décrits en Afrique du Nord et dans les pays développés.

### Contribution de notre étude à la connaissance

- Les particularités épidémio-cliniques en Afrique Subsaharienne;
- Les particularités de la sémiologie échographique des MH.
- La comparaison des résultats d'échographie à ceux de l'anatomopathologie.

## Conflits d’intérêts

Les auteurs ne déclarent aucun conflit d’intérêts.
